# Genome-wide analysis provides a deeper understanding of the population structure of the *Salmonella enterica* serotype Paratyphi B complex in Bangladesh

**DOI:** 10.1099/mgen.0.000617

**Published:** 2021-09-22

**Authors:** Sadia Isfat Ara Rahman, Alyce Taylor-Brown, Farhana Khanam, Ashraful Islam Khan, Gal Horesh, Zoe A. Dyson, Yasmin Ara Begum, Emran Kabir Chowdhury, Firdausi Qadri, Gordon Dougan, Nicholas R. Thomson

**Affiliations:** ^1^​ Infectious Diseases Division, International Centre for Diarrhoeal Disease Research, Bangladesh (icddr,b), Dhaka, Bangladesh; ^2^​ Wellcome Sanger Institute, Wellcome Genome Campus, Hinxton, Cambridge, UK; ^3^​ Department of Medicine, University of Cambridge, Cambridge, UK; ^4^​ London School of Hygiene and Tropical Medicine, London, WC1E 7HT, UK; ^5^​ Department of Infectious Diseases, Central Clinical School, Monash University, Melbourne, Victoria 3004, Australia; ^6^​ Department of Biochemistry and Molecular Biology, University of Dhaka, Dhaka, Bangladesh

**Keywords:** *Salmonella *Paratyphi B, serotyping, surveillance, whole-genome sequencing, enteric disease

## Abstract

The *Salmonella enterica* serotype Paratyphi B complex causes a wide range of diseases, from gastroenteritis to paratyphoid fever, depending on the biotypes Java and *sensu stricto*. The burden of Paratyphi B biotypes in Bangladesh is still unknown, as these are indistinguishable by *Salmonella* serotyping. Here, we conducted the first whole-genome sequencing (WGS) study on 79 *Salmonella* isolates serotyped as Paratyphi B that were collected from 10 nationwide enteric disease surveillance sites in Bangladesh. Placing these in a global genetic context revealed that these are biotype Java, and the addition of these genomes expanded the previously described PG4 clade containing Bangladeshi and UK isolates. Importantly, antimicrobial resistance (AMR) genes were scarce amongst Bangladeshi *S*. Java isolates, somewhat surprisingly given the widespread availability of antibiotics without prescription. This genomic information provides important insights into the significance of *S*. Paratyphi B biotypes in enteric disease and their implications for public health.

## Data Summary

The genome sequence data generated in this study have been deposited at the European Nucleotide Archive (ENA) under accession numbers ERR4339057–ERR4619485 (accession numbers and metadata available in Tables S1 and S2, available in the online version of this article). Custom R and Python scripts used for comparative pan-genome analysis are available at https://github.com/ghoresh11/Salmonella_ParaB.

**Impact Statement**

*
Salmonella enterica* serotype Paratyphi B complex (*S*. Paratyphi B complex) has long been a source of confusion for microbiologists, as the two biotypes in this serotype have until now been indistinguishable by *Salmonella* serotyping. Further, there is still very little molecular information available to understand the population structure of the *S*. Paratyphi B complex in many regions. In 2016, Connor *et al.* reported the utility of whole-genome sequencing (WGS) to distinguish this serotype into two biotypes, *sensu stricto* and Java, which cause, respectively, paratyphoid fever and gastroenteritis. Our study is the first to apply genomics to the *S*. Paratyphi B complex in a hospital-based surveillance study in sites across Bangladesh, where WGS analysis classified these serotyped Paratyphi B as biotype Java, associated with diarrhoeal symptoms. This study reiterates the advantage of WGS studies in addition to molecular and phenotypic methods.

## Introduction

The genus *Salmonella *, which belongs to the family *Enterobacteriaceae *, is commonly associated with bacterial foodborne illness in developed countries. The species *Salmonella enterica* consists of several subspecies, the first of which, *S. enterica* subspecies enterica, is commonly split into typhoidal and non-typhoidal *Salmonella* (NTS), based on the disease syndrome [1]. The *Salmonella enterica* serotype Paratyphi B complex (*S*. Paratyphi B complex) causes both potentially life-threatening invasive paratyphoid fever and non-invasive gastroenteritis; both typhoidal and non-typhoidal types share the same somatic O antigen formula (1,4,[5],12 with the b:1,2-type of flagellar H antigen) [2], resulting in a point of confusion for microbiologists. This serotype has been further subdivided into two biotypes based on the ability to ferment dextrorotatory tartrate (*d*Ta) and to form a slime wall: *Salmonella enterica* serotype Paratyphi B variant *sensu stricto* (*S*. Paratyphi B *sensu stricto; d*Ta^−^, slime wall-positive) and *Salmonella enterica* serotype Paratyphi B variant Java (*S*. Java; *d*Ta^+^; slime wall-negative), which collectively comprise the *S*. Paratyphi B complex [2–4].

Whilst the *d* -tartrate reaction is used clinically to distinguish these biotypes, it can be unreliable and provides no phylogenetic resolution. Therefore, isolates of the serotype *S*. Paratyphi B complex have been further subtyped by phage typing [5], IS*200* profiling [6], multilocus sequence typing (MLST) [7], clustered regularly interspaced short palindromic repeats (CRISPR) typing [8] and also whole-genome sequencing (WGS). In a recent study, WGS analysis revealed that the *S*. Paratyphi B complex is represented by 10 distinct lineages (phylogroups; PGs), based on the core gene phylogeny [2]: the invasive *S*. Paratyphi B *sensu stricto* (*d*Ta^−^) isolates grouped into a single lineage (PG1), while the remaining PGs comprised diverse lineages of biotype *S*. Java (*d*Ta^+^). However, the pathogenic properties of *S*. Paratyphi B *sensu stricto* and *S*. Java remain poorly understood especially in low-income settings, like Bangladesh.

There has been an increase in reports of *S*. Java infections, especially from poultry sources, observed in Germany, the Netherlands and Belgium since 1990 [9] and in the UK since 2010 [10]. In addition, non-European countries such as Saudi Arabia [11] and Bangladesh [12] have also noted an increasing incidence of *S*. Java in poultry farms. In Bangladesh, very few data exist on the prevalence and incidence of *S*. Paratyphi B compared to other typhoidal *Salmonella* such as *S*. Typhi [13] and *S*. Paratyphi A. To provide a genomic snapshot of the *S*. Paratyphi B complex in Bangladesh, we sequenced 79 *Salmonella* isolates previously serotyped as Paratyphi B in nationwide hospital-based enteric disease surveillance in Bangladesh between 2014 and 2018. This is the first WGS-based study characterizing the genetic diversity of *S*. Paratyphi B isolates causing diarrhoeal disease in Bangladesh.

## Methods

### Ethics statement

Ethical approval was obtained from the Research Review Committee (RRC) and Ethical Review Committee (ERC) of the International Centre for Diarrhoeal Disease Research, Bangladesh (icddr,b) (reference number PR#12060). Informed written consent was taken from adult participants and the legal guardians of child participants under 18 years old.

### Study settings, sample collection and bacteria isolation

**Fig. 1.**
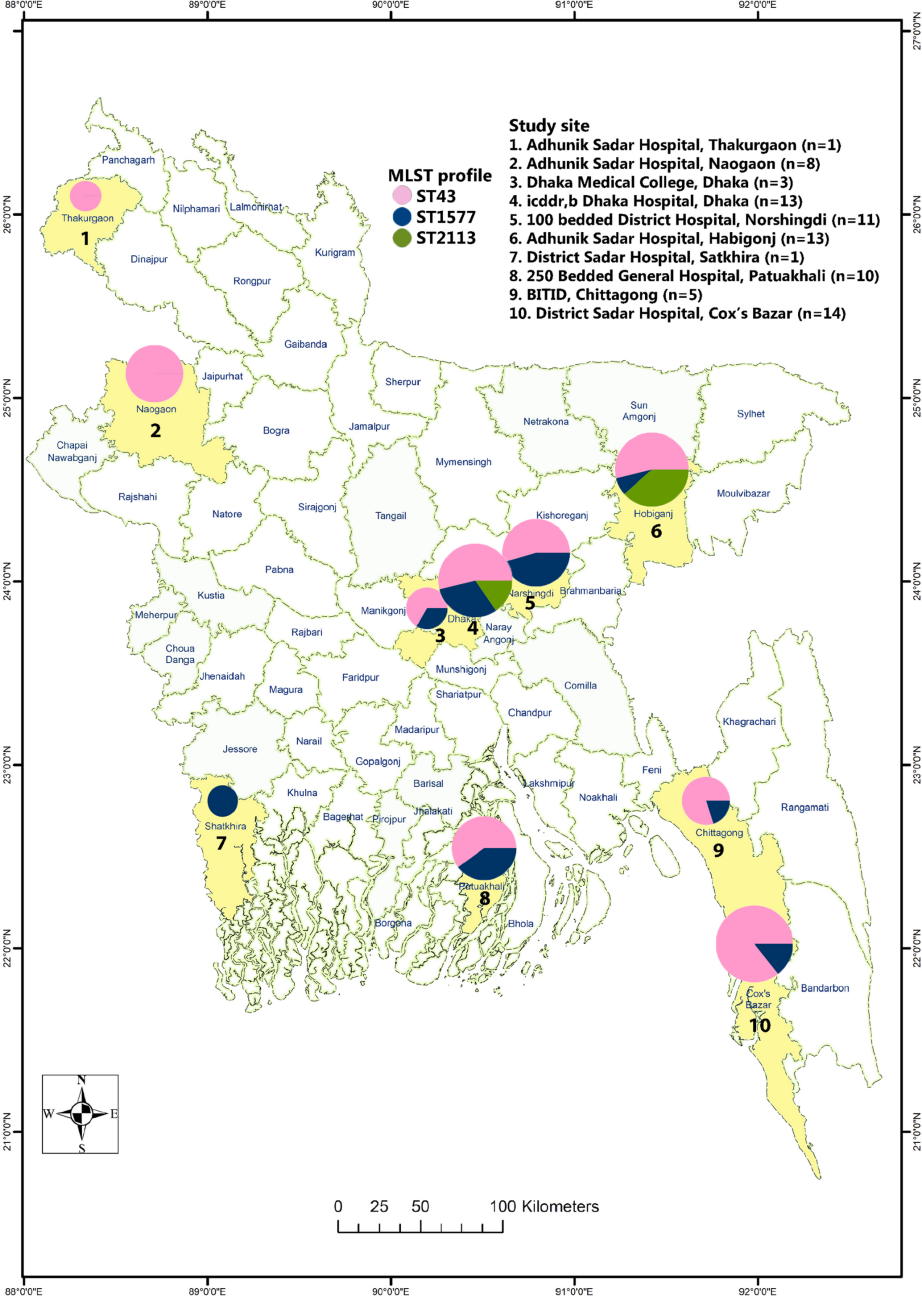
Map of nationwide study surveillance sites in Bangladesh, June 2014–June 2018. Pie charts at each site depict the MLST distribution. The number of *S*. Java-positive cases (*n*) at each study site is also shown in the key.

This study utilized samples collected from an established nationwide enteric disease surveillance system being carried out in 10 hospitals across 8 divisions of Bangladesh in a collaboration between the Institute of Epidemiology, Disease Control and Research (IEDCR) and icddr,b (Fig. 1, Table 1). The surveillance sites were selected based on reports of acute watery diarrhoea according to the national District Health Information Software v2 Database from Directorate General of Health Services (DGHS) [14, 15]. It is a large, longitudinal, multi-pathogen surveillance study that included diarrhoeal patients infected with a variety of enteric pathogens: *Vibrio cholerae *, ETEC, *Shigella *, and typhoidal and non-typhoidal *Salmonella *. Patients were enrolled into the enteric disease surveillance study if they were over 2 months old, and attended hospital with either (a) loose or liquid stools ≥3 times; (b) loose or liquid stools causing dehydration <3 times; or (c) at least one bloody loose stool in the previous 24 h [14, 15]. Demographic and clinical information, including age, gender, date of illness onset and date of sample collection, was obtained from each participant (Tables 1 and S1).

**Table 1.** Prevalence of *S*. Paratyphi B (*n*=107) collected from 10 hospital-based enteric surveillance sites in Bangladesh from June 2014 to June 2018

Study site	Division	Enrolled diarrhoeal patients, *n*	*S.* Paratyphi B-positive, *n* (%)
100 bedded District Hospital, Narshingdi	Dhaka	1156	13 (1.12)
Dhaka Medical College, Dhaka	Dhaka	737	4 (0.54)
icddr,b* Hospital, Dhaka	Dhaka	14889	31 (0.21)
District Sadar Hospital, Cox’s Bazar	Chittagong	2089	14 (0.67)
BITID†, Chittagong	Chittagong	1860	8 (0.43)
Adhunik Sadar Hospital, Naogaon	Rajshahi	1605	10 (0.62)
Adhunik Sadar Hospital, Thakurgaon	Rangpur	1715	1 (0.06)
Adhunik Sadar Hospital, Habigonj	Sylhet	2247	14 (0.62)
250 Bedded General Hospital, Patuakhali	Barisal	1805	10 (0.55)
District Sadar Hospital, Satkhira	Khulna	1434	2 (0.14)
Total		29 537	107 (0.36 %)

*icddr,b; International Centre for Diarrhoeal Disease Research, Bangladesh.

†BITID; Bangladesh Institute of Tropical and Infectious Diseases.

Stool samples collected from individuals exhibiting diarrhoeal symptoms were cultured by streaking on MacConkey agar and *Salmonella–Shigella* (SS) agar. After overnight incubation at 37 °C, non-lactose-fermenting colonies were inoculated for biochemical testing and those showing typical characteristics of *Salmonella* spp. were serotyped using *Salmonella *-specific somatic O and flagellar H antiserum (Denka Sieken Tokyo, Japan) [16] for confirmation of *S*. Paratyphi B.

### DNA extraction, WGS and dataset compilation

Genomic DNA was extracted from the *S*. Paratyphi B strains using the Wizard Genomic DNA kit (Promega, Madison, WI, USA) according to the manufacturer’s instructions for genomic analysis. WGS was performed at the Wellcome Sanger Institute (WSI) using the Illumina Hiseq 2500 platform (Illumina, San Diego, CA, USA) to generate 150 bp paired-end reads. Sequence data quality was assessed using FastQC (http://www.bioinformatics.babraham.ac.uk/projects/fastqc).

To provide global context for the Bangladeshi *S*. Paratyphi B genomes, 180 *S*. Paratyphi B complex genomes from Connor *et al.* [2] and 12 *S*. Paratyphi B *sensu stricto* genomes from patients presenting with enteric fever symptoms from Higginson *et al.* [4] were also included in this study (Table S2).

### Read alignment and SNP calling

Illumina reads for all 271 genomes were mapped against the reference *S*. Paratyphi B strain SPB7 (accession number CP000886) using SMALT v0.7.4 [17], with PCR duplicate reads flagged using Picard v1.92 (http://broadinstitute.github.io/picard). Candidate single-nucleotide polymorphisms (SNPs) relative to the reference having a quality score >30, consensus base quality >20 and read depth >5 were identified using SAMtools [18] and were extracted using SNP-sites [19]. SNPs called in prophage regions and repetitive sequences, or in recombinant regions as detected by Gubbins (v2.3.2) [20], were excluded, resulting in a final SNP alignment of 132 593 bp for the 271 *S*. Paratyphi B complex genomes.

### Phylogenetic, population genetic analysis and statistical analyses

Maximum-likelihood (ML) phylogenetic trees were inferred from the SNP alignments using RAxML (v8.2.8) [21]. A generalized time-reversible model and a gamma distribution were used to model site-specific rate variation (GTR+ Γ substitution model; GTRGAMMA in RAxML) with 100 bootstrap pseudoreplicates used to assess branch support for the ML phylogeny. SNP alleles from PG10 isolates reported in Connor *et al.* [2] were included as an outgroup to root the tree. The resulting phylogenies were visualized and annotated using FigTree (available at: http://tree.bio.ed.ac.uk/software/figtree), iTOL [22] and the R package ggtree [23].

We performed hierarchical Bayesian Analysis of Population Structure (BAPS) implemented in RhierBAPS [24] to redefine the *S*. Paratyphi B subpopulation structure.

To determine the statistical relationships between sequence type distribution and epidemiological factors, we conducted Fisher’s exact tests implemented in STATA [25].

### *De novo* genome assembly, annotation and comparative pan-genome analysis

Raw sequence reads were assembled *de novo* using Unicycler (v0.3.0b) [26] and annotation was performed by PROKKA (v1.5) [27]. The quality of genome assemblies were assessed using QUAST (v5.0.2) [28] and the detailed quality reports are summarized in Table S1. The pan-genome was determined with Roary [29] from the annotated assemblies, using a blastp percentage identity of 95 % and a core definition of 95 % of the included isolates. To estimate the openness of the pan-genome(s), we used the Heaps function within the Micropan R package [30], which calculates the curve fit constant according to Heaps’ law [31]: n=k*N^−α^, where *n* is pan-genome size, *N* is the number of genomes and *k*, *γ* are curve-specific constants [32]. The curve specific constant, *α*=1−*γ* determines whether the pan-genome of a bacterial variant (e.g. species, biotype or lineage) is closed (*γ*<0, *α*>1) or open (0<*γ*<1, *α*<1).

Comparative pan-genome analysis, using custom R and Python scripts available at https://github.com/ghoresh11/Salmonella_ParaB, was performed to identify biotype- and clade-specific genes. The frequency of each gene in the pan-genome amongst all *S*. Paratyphi B *sensu stricto* and amongst all *S*. Java isolates was calculated. Similarly, the frequency of all genes in BAPS cluster 1.1 (PG3/PG4 clades) relative to the rest of the clades were calculated. A gene was defined as core and specific to a biotype or clade if it was present in more than 95 % of one biotype/clade and absent from more than 95 % of the isolates of the other biotype/clade. To investigate the synteny in the loci containing the biotype- or clade-specific genes, a synteny graph similar to that presented elsewhere [33] was constructed in the regions of interest. A region was defined by the two flanking genes that were consistently identified upstream of and downstream to the biotype/clade-specific loci. A graph was constructed from the annotation files such that each node in the graph is a gene, and the weighted edge between two genes represents the number of times they were adjacent to each other across all genomes. The results of these analyses were visualized using Phandango [34] and Cytoscape [35]. In addition, we used the basic local alignment search tool (blast) [36] to identify the distribution of group-specific genes throughout the species, InterProScan (v5) [37] to predict the function of the group-specific hypothetical proteins and EffectiveDB [38] to predict secreted proteins.

### Antimicrobial resistance (AMR) gene detection, plasmid detection, virulence factor detection, *in silico* serotype prediction and MLST analysis

We detected AMR genes and plasmid replicons using ARIBA [39] in conjunction with the comprehensive antibiotic resistance database (CARD) [40] and the PlasmidFinder [41] database, respectively. We used the same approach to detect virulence factors, using the Virulence Factor Database (VFDB) [42]. The Salmonella In Silico Typing Resource (SISTR) [43], implemented in PathogenWatch [44], was used for *in silico* serotype prediction of the sequenced genomes. The mapping-based allele typer SRST2 [45] was used to assign sequence types (STs) to each genome according to the *S. enterica* MLST database.

## Results

### Demographic and clinical characteristics of *S*. Paratyphi B strains isolated from diarrhoeal patients and their genome assembly metrics

The goal of this study was to investigate the prevalence and genomic diversity of *S*. Paratyphi B complex in Bangladesh. A total of 29 537 diarrhoeal patients presenting to 1 of 10 sentinel surveillance sites were enrolled into this study between June 2014 and June 2018 (see the Methods section for further details). Of these patients, 0.36 % (107/29 537) were confirmed as *Salmonella enterica* serotype Paratyphi B-positive by serotyping, with the antigenic formula O1,4,5,12:Hb:1,2. The percentage of patients presenting with *S*. Paratyphi B at each surveillance site was low, ranging from 0.06–1.12 %, compared to other enteric infections (for example the equivalent range for *Vibrio cholerae* was 1.10–18.3 % of patients [14]) (Table 1). The Narshingdi district hospital in the Dhaka division had the highest percentage of *S*. Paratyphi B-positive patients (1.12 %) in this study.

Of the 107 *S*. Paratyphi B-positive samples, only 79 *S*. Paratyphi B strains were available for sequencing in this study. Sequencing of these genomes produced assemblies containing on average 36.39 contigs (≥1000 bp) and the total assembly lengths (consisting of contigs ≥1000 bp) ranged from 4 619 574 to 4 792 431 bp (an average of 4 664 961 bp); the expected size for *S*. Paratyphi B genomes. The mean of scaffold N50 sizes was 375 447 bp (range 289 235 to 399 505 bp) (Table S1). For our downstream analysis we utilized all contigs over 1000 bp in length.

Patient metadata including age and sex data as well as clinical symptom data were available for most patients (Tables 2 and S1). Among these, 63 patients were adults (17–85 years of age; median age 31 years; Table 2), with 39 of these aged between 17 and 35, and 11 were young children (≤5 years old). The majority of the patients were female (*n*=50; 68 %). Loose watery stool (LWS) was reported more frequently, with a longer duration of diarrhoea (*n=*43 with average duration 2.61 days), than rice watery stool (RWS) (*n*=33 with average duration 1.72 days). The most common combination of clinical symptoms, recorded in 12 patients, was RWS with vomiting, some or severe dehydration and abdominal cramping. No bloody diarrhoea was reported among the Bangladeshi *S*. Paratyphi B-positive population (Table 2). We did not observe any co-infections with any other enteric pathogens targeted in the surveillance study.

**Table 2.** Demographic and clinical characteristics of *S*. Paratyphi B-positive patients (*n*=79) in this study

Characteristics	*S*. Paratyphi B-positive, *n* (%)
**Demographic factors**	
Age (years)*	
0–5	11 (14.86)
17–85	63 (85.14)
Median age of patients (IQR)	31 (24.22)
Sex†	
Male	23 (31.51)
Female	50 (68.49)
**Clinical factors**	
Stool nature‡	
Loose watery	43 (55.84)
Rice watery	33 (42.85)
Bloody	0 (0.0)
Formed	1 (1.29)
Dehydration status‡	
None	14 (18.18)
Some	48 (62.33)
Severe	15 (19.48)
Abdominal cramp§	
Yes	47 (67.14)
No	23 (32.85)
Vomiting‡	
Yes	57 (74.02)
No	20 (25.97)
Duration of diarrhoea (days)||	
No diarrhoea	6 (9.09)
1	19 (28.79)
2	23 (34.85)
3	12 (18.18)
4	3 (4.55)
5	3 (4.55)

*This information was available for 74 patients.

†This information was available for 73 patients.

‡This information was available for 77 patients.

§This information was available for 70 patients.

||This information was available for 66 patients.

### Population structure of the *S*. Paratyphi B complex in Bangladesh

**Fig. 2.**
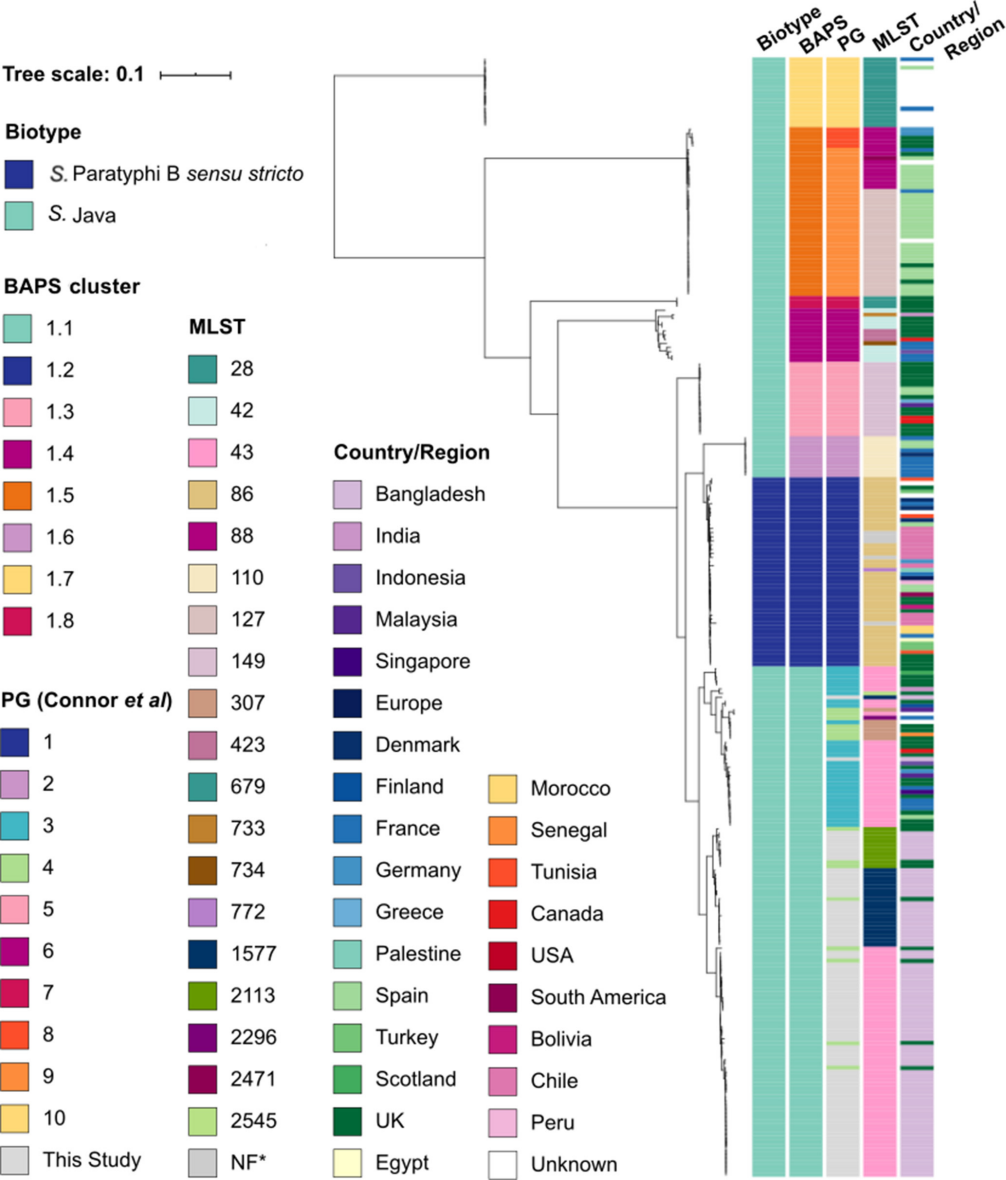
Maximum-likelihood outgroup-rooted phylogenetic tree of 271 *S*. Paratyphi B strains from the global collection, including Bangladeshi *S*. Java isolates from this study. Whole-genome SNP tree with recombination regions removed and outgroup rooted with PG10/BAPS1.7. The coloured strips show the biotype, BAPS cluster (this study), PG (Connor *et al*. [2]), MLST and country or region of isolation for each isolate; see colour legend. The tree scale bar indicates the estimated mean number of nucleotide substitutions per site.

To further classify the biotype of the isolates serotyped as *S.* Paratyphi B in the surveillance study, and investigate the phylogenetic relationships between the Bangladeshi and global isolates belonging to the *S*. Paratyphi B complex, we constructed a global phylogeny which included 192 contextual *S*. Paratyphi B complex genomes originating from over 20 countries [2, 4] and the 79 Bangladeshi genomes sequenced in this study (Fig. 2). Previously, Connor *et al*. reported that PG1 comprised *S*. Paratyphi B *sensu stricto*, while *S*. Java genomes were represented by PG2 to PG10 [2]. Our WGS data revealed that all 79 Bangladeshi isolates serotyped as *S*. Paratyphi B were classified as biotype Java. This is consistent with the clinical data, which showed that all isolates were taken from patients presenting with non-invasive diarrhoeal disease (Table 2). Our genomes clustered within two of the previously described *S*. Java clades; either PG3 (*n*=2) or PG4 (*n*=77) in the previously published global phylogeny [2], with up to 4709 SNPs separating genomes in the two clades (median SNP distance of 2850 bp).

Furthermore, the Bangladeshi *S*. Java isolates contributed substantially to an expansion of the known PG4 diversity, which originally mainly comprised *S*. Java isolates originating from the UK. PG3, on the other hand, only contained two Bangladeshi *S*. Java genomes, with the remainder of the clade comprising isolates from the UK, continental Europe, the USA, and South and Southeast Asia (Fig. 2). Whilst our genomes fall within the previously defined PGs, the addition of our 79 Bangladeshi *S*. Java isolates to the published phylogeny disagreed with the original definition of phylogroups PG3 and PG4 by Bayesian hierarchical clustering, which in our updated phylogeny, merged PG3 and PG4 into a single cluster at BAPS level 1 (ascribed BAPS cluster 1.1) (Fig. 2). Potential reasons for this discrepancy are noted in the Discussion.

**Fig. 3.**
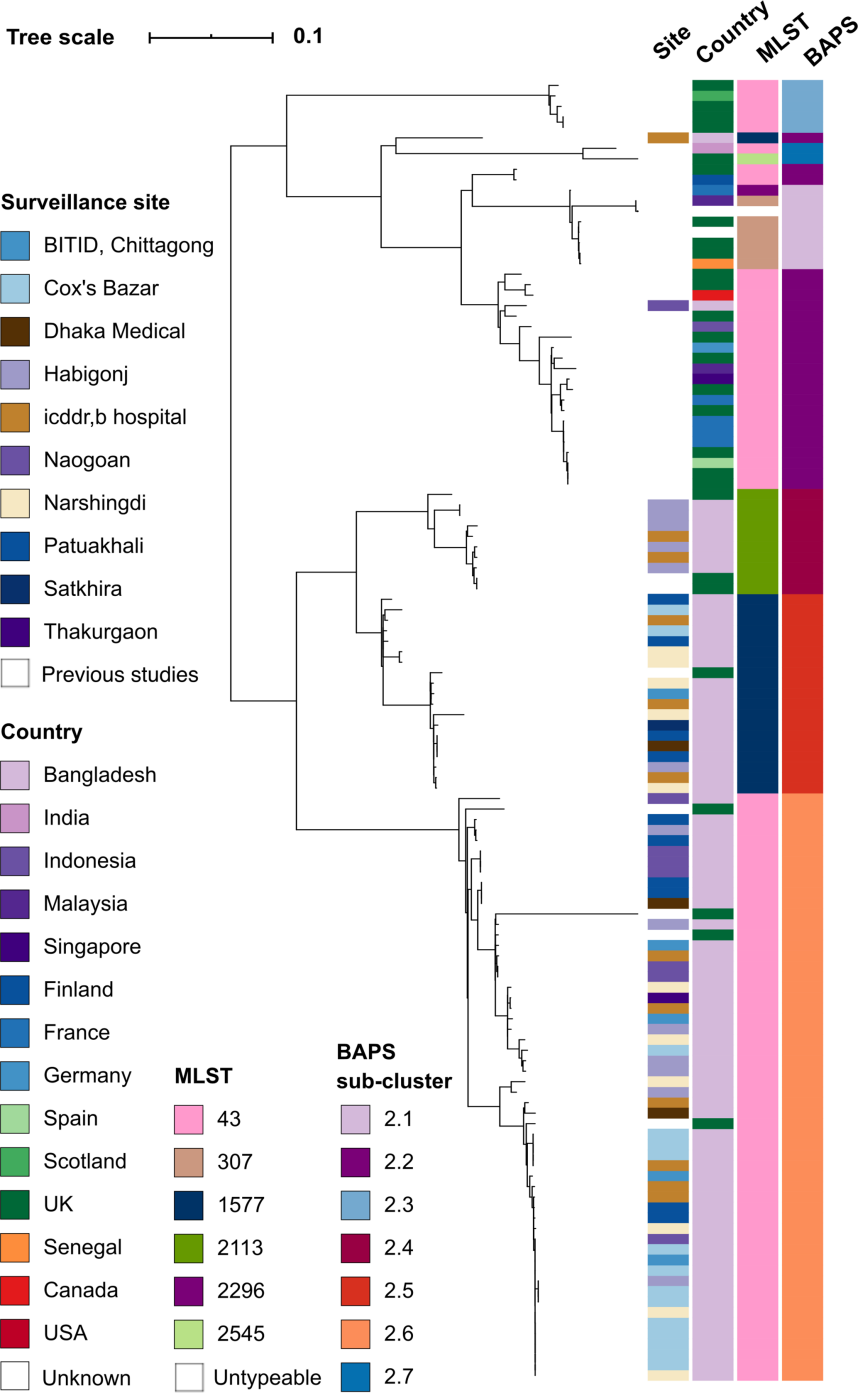
Mid-point-rooted maximum-likelihood phylogeny of *S*. Java cluster 1.1 (PG3-4). Whole-genome SNP tree with recombination regions removed and mid-point rooted. The coloured strips alongside the tree show the surveillance site, country or region of isolation, MLST and BAPS sub-cluster for each isolate; see colour legend. The tree scale bar indicates the estimated mean number of nucleotide substitutions per site.

To analyse the Bangladeshi *S*. Java population structure in finer detail, we constructed a phylogeny of the *S*. Java isolates belonging to BAPS cluster 1.1 (*n*=123) from 20908 chromosomal SNPs across the whole genome (Fig. 3). This revealed that the population structure of *S*. Java in Bangladesh is characterized by three STs: ST43 (*n*=53, 67.1 %), ST2113 (*n*=19, 24.1 %) and ST1577 (*n*=7, 8.9 %) (Fig. 1). ST43 is a globally distributed ST, while STs 2113 and 1577 have only been detected in the UK and Bangladesh; the former was detected for the first time in Bangladesh in this study (Fig. 3).

We then defined seven sub-clusters at BAPS level 2: PG3 isolates grouped into sub-clusters 2.2, 2.3 and 2.7 and PG4 grouped into 2.1, 2.4, 2.5 and 2.6. BAPS sub-clusters 2.6, 2.5 and 2.4 corresponded with STs 43, 2113 and 1577, respectively (Fig. 3). We did not observe any phylogeographical clustering of isolates within Bangladesh, nor was there a significant difference between the ST distributions in the Dhaka sites combined (*n*=3 sites) relative to their distribution in the rest of the regions in Bangladesh (*n*=7 sites) (*P*=0.121, Fisher’s exact test) (Fig. 1). Only two hospital sites (icddr,b hospital and Habigonj district hospital) harboured all three STs. ST43 was distributed throughout all study sites in Bangladesh except Satkhira, from which only one isolate was obtained. We did not observe any significant association between age group (children, adult) or sex (male, female) and the distribution of *S*. Java STs, respectively, (*P*=0.361 and *P*=0.469, using Fisher’s exact test) (Table S1).

Examining ST distribution in the context of clinical characteristics, isolates typed as ST2113 and ST1577 were commonly associated with patients with LWS (*n*=7, 100 % and *n*=13, 72 %, respectively). Whereas, LWS and RWS were reported at similar frequencies (*n*=22 and *n*=28, respectively) for ST43 isolates. Of note, among the 15 cases with severe dehydration, 73 % (*n*=11) were associated with ST43. Furthermore, the duration of diarrhoea differed throughout the surveillance sites: for example, the average duration of diarrhoea in Habigonj was 2.83 days (*n*=12) compared to only 1.43 days (*n*=14) in Cox’s Bazar (Table S1).

We explored the distribution of virulence factors (VFs) throughout the Bangladeshi *S*. Java genomes in relation to the clinical characteristics and the BAPS sub-clusters. A detailed list of the virulence genes detected in a total of 271 genomes is summarized in (Table S3). RWS was observed more frequently in BAPS sub-cluster 2.6 than the other sub-clusters (*n*=28 and 5, respectively; Table S1). Interestingly, the genomes in this sub-cluster harboured the *tcfABCD* genes, which were absent from all other lineages except for PG7 (Fig. S1). These genes encode the Typhi colonization factor and are also present in other NTS serovars [46, 47]. Interestingly, these same genomes lacked the fimbrial *stfACDEFG* genes [48], which were present in all other lineages except for PG6. We did not identify VFs that were specific to Bangladeshi isolates, and all other differences we observed between the lineages were reported by Connor *et al.* [2].

### Comparative pan-genome analysis

**Fig. 4.**
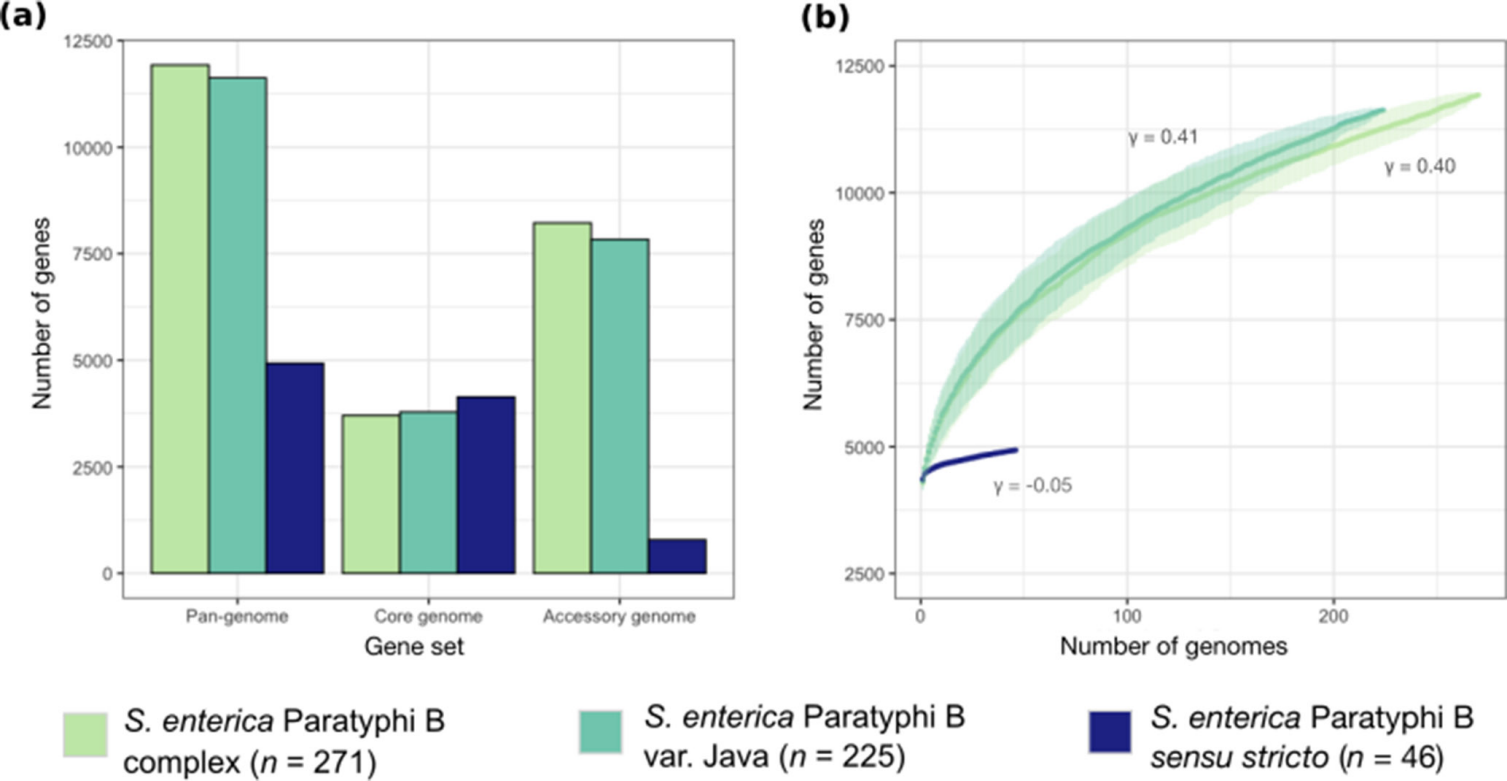
Pan-genome dynamics of the *S*. Paratyphi B complex. (**a**) The pan, core and accessory genomes and (**b**) gene accumulation curves are depicted for the *S*. Paratyphi B complex and each biotype; see key for colour. Here, core genes are defined as genes present in ≥95 % of strains and accessory genes are present in <95 % of strains. Error bars above and below the median are depicted by shading above and below the curve in (**b**).

To investigate gene distribution among the *S*. Paratyphi B complex biotypes, we conducted a core- and pan-genome analysis on all 271 genomes. This revealed that 11 929 genes comprised the *S*. Paratyphi B complex pan-genome (271 genomes; PG1-10), with 3706 genes in the core genome (present in ≥95 % of genomes) and 8223 in the accessory genome (present in <95 % of genomes). Further, the pan-genome sizes of *S*. Paratyphi B *sensu stricto* (46 genomes; PG1) and *S*. Java (225 genomes; PG2-10) were 4930 and 11 625 genes, respectively. Within these, we determined 4141 core and 789 accessory genes for *S*. Paratyphi B *sensu stricto* and 3787 core and 7838 accessory genes for *S*. Java (Fig. 4a).

The gene accumulation curve for the *S*. Paratyphi B complex, carried out in accordance with Heaps’ law [31, 32] (see Methods section) is driven by the diversity within *S*. Java. This is demonstrated by similar curve fitting parameter values for the complex and *S*. Java of *γ*=0.40 and *γ*=0.41, respectively, while that of *S*. Paratyphi B *sensu stricto* is much lower (*γ*=−0.05) (Fig. 4b). This suggests that within the *S*. Paratyphi B complex, the pan-genome of *S*. Java remains open and, as more strains are sequenced, new genes will be identified among these organisms, while the gene accumulation curve of *S*. Paratyphi B *sensu stricto* converged rapidly and is approaching closed.

To investigate gene flux within the *S*. Paratyphi B complex, we identified biotype-specific genes and loci (Table S4). We defined a core and specific gene as one present in ≥95 % of genomes of one group and absent in >95 % of the other group. Based on these criteria, we identified 20 core and specific genes for *S*. Paratyphi B *sensu stricto* (Fig. S2a) and 30 core and specific genes for *S*. Java (Fig. S2b). We confirmed that the *S*. Paratyphi B *sensu stricto*-specific genes form a single gene block, at the same locus. This gene block was assembled on the same contig in ≥93 % of genomes and split over different contigs in the remaining ~7 % of genomes. On the other hand, the *S*. Java-specific genes are clustered at three genetic loci (in ≥99 % of genomes).

The three *S*. Java-specific loci are located within a 181 CDS-long chromosomal region and differ in length (Fig. S3). The first of these loci contains only a single 1134 bp gene (hypothetical protein; group_270; *S *. enterica serotype Paratyphi B str SPB7 v1 locus tag 01289), predicted to encode a hypothetical protein with a carboxymuconolactone decarboxylase (CMD) domain that may have peroxidase activity. The gene is positioned between *rnb* and *fabI* (Figs S2a and S4a). Given its proximity to the SPI-2 effector *steC*, we ran this gene through T3SS effector prediction software, which predicted that the gene product may be secreted.

The second *S*. Java-specific locus carried genes predicted to encode the ABC transporters *ArtM*, *YecS* and *FliY* (also referred to as *TcyJ*), involved in amino acid transport, as well as the transcriptional repressor *FrmR* and the SPI-2 type III secretion system effector protein *SseJ* (Figs S2a and S4b). Homologues of some of these genes, excluding *sseJ*, are found at other distinct, conserved, loci in *S*. Paratyphi B *sensu stricto*, suggesting that these genes are not essential for pathogenicity and have been lost by *S*. Paratyphi B *sensu stricto*. Interestingly, synteny analysis showed that in BAPS 1.7 (PG10), a primarily animal-associated clade, one of the hypothetical proteins (group_1829) has been replaced by group_2748 (Fig. S4b).

The final locus encodes numerous hydrolase and oxidoreductase enzymes involved in metabolism of amino acids, carbohydrates and nitric oxide (*norV, glsA, gabD, sad, gutB, yjjL, cbh, hoxK*) and the *hyaABC* genes and their chaperones (Figs S2b and S4c), which may facilitate the ability to utilize locally generated hydrogen. Hence, this locus encodes several genes involved in tolerance to stressors often associated with the gut niche and also encoded on this locus are outer-membrane protein (*ompC*) and tetracycline resistance gene (*tetA*). Although the genes in this locus were absent from the *S*. Paratyphi B *sensu stricto* genomes (Figs S2b and S3), blast analysis revealed that some genes in this group have homologues in other *Salmonella* serovars, including *S*. Enteritidis, *S*. Typhimurium and *S*. Kentucky. This suggests they have potentially been lost by *S*. Paratyphi B *sensu stricto,* rather than gained by *S*. Java. Moreover, this locus is flanked at one end by the non-coding RNA STnc560, includes STnc170 and the Hfq binding RNA *isrF*, and has a selenocysteine insertion sequence SECIS_3 upstream. The diversity observed with respect to the gene arrangement at this locus further supports the gene loss hypothesis, with clade-specific variations noted (Fig. S4c).

The *S*. Paratyphi B *sensu stricto*-specific locus appears to be a bacteriophage/prophage, spanning approximately 44 000 bp, containing approximately 59 genes (Fig. S2a, Table S4), many of which have phage-related annotations, while others are predicted to encode hypothetical proteins. The borders of this locus are difficult to define as (a) the gene order is not conserved in all genomes, (b) assemblies are fragmented in this region and (c) some genes within this region have homologues in other clades. Among numerous hypothetical proteins in this locus are bacteriophage-related genes such as the bacteriophage Mu F-like protein, proposed to be involved in viral capsid assembly. This locus also encodes SopE, which is a SPI-2 secreted effector protein also encoded by *S*. Typhi that induces nitric oxide synthetase (iNOS) in the host intestine, leading to inflammation [49]. It has been shown that this effector can be transferred between unrelated phages associated with different serovars [50]. No other SPI-2 effectors appear to be missing from the *S*. Java genomes. Further, blast analysis of the ~33 000 bp region on the forward strand flanked by *dicA* and *sopE* revealed a high nucleotide identity to *S *. enterica serovar Typhi genomes (94–97 % across 51–62 % of the query region). A homologue of *sopE*, *sopE2*, which activates a different set of Rho GTPases to SopE, is encoded in the majority of Spanish isolates in BAPS cluster 1.5 (PG9), but none of the Bangladeshi isolates. This gene is also encoded by *S*. Typhimurium.

A second locus was found to be specific to *S*. Paratyphi B *sensu stricto* (BAPS cluster 1.2; PG1) and its close relative, BAPS cluster 1.6 (PG2) (Fig. S1a). This locus is predicted to encode some sugar metabolizing and transport enzymes (*gutB, yggF, cmtB, mtlA*) and provides evidence of compensatory mechanisms with respect to the sugar metabolizing enzymes in the *S*. Java-specific gene set.

Using this same approach, we identified five genes that were specific and core for BAPS cluster 1.1 (containing all the Bangladeshi isolates), relative to all other BAPS level 1 clusters. One of the predicted genes, the SPI-2 effector *sseI*, is located in a small gene cluster with a hypothetical protein and a transposase. The other two genes appear to be associated with a clade-specific phage that is also present in PG7, and/or some of PG6, mostly in isolates from the UK.

### AMR and plasmid profiles of *S*. Paratyphi B complex

**Fig. 5.**
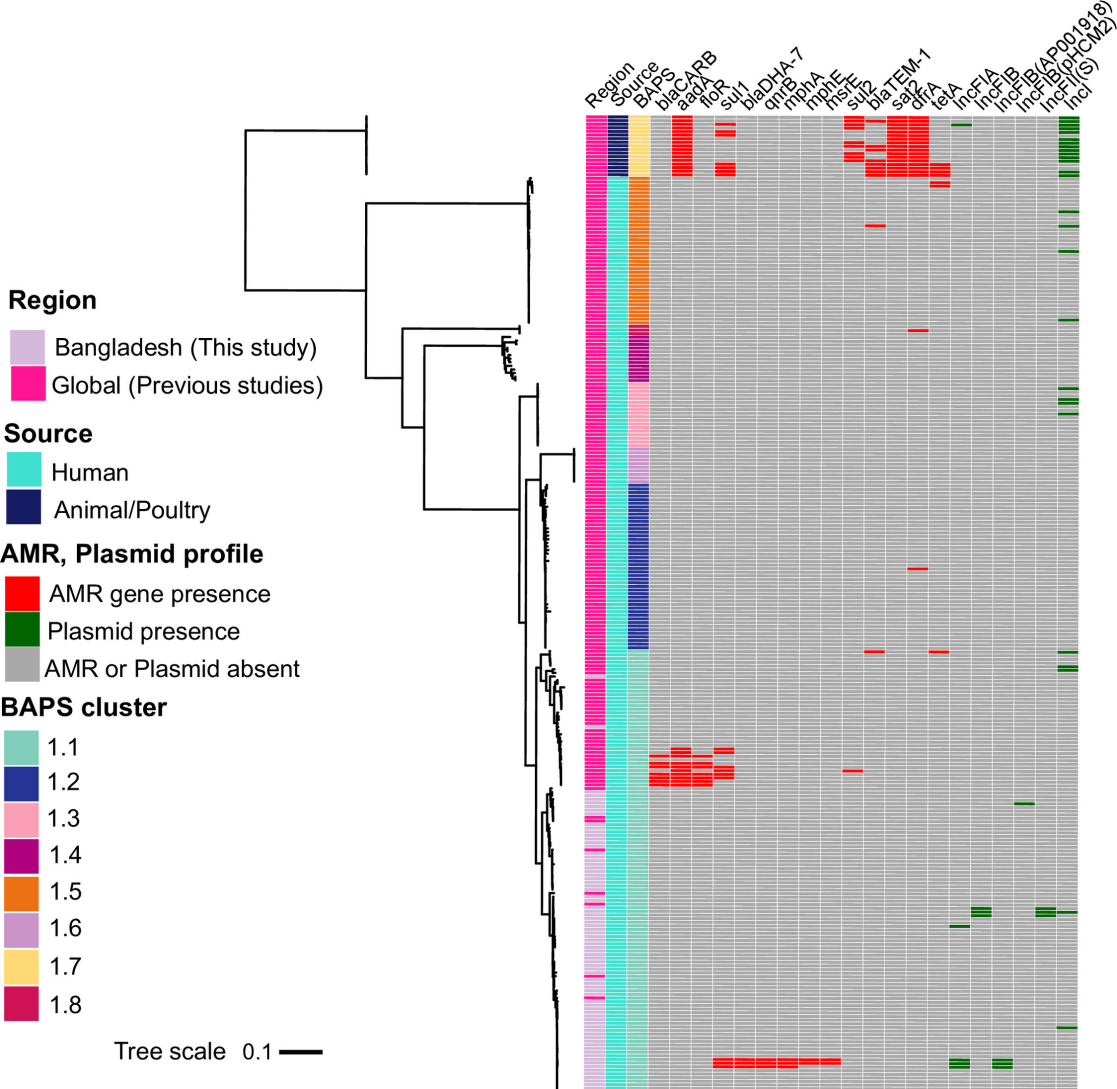
Antimicrobial resistance gene and plasmid replicon distribution among the *S*. Paratyphi B complex. A maximum-likelihood outgroup-rooted tree of 271 strains from the global collection, including Bangladeshi *S*. Java isolates from this study, alongside a presence/absence matrix of AMR genes and plasmid replicons for each isolate. Only genes that were differentially detected between global and Bangladeshi human-associated *S*. Java (BAPS cluster 1.1), or global human- and animal-associated global *S*. Java (BAPS cluster 1.7), are shown, in order to observe the differences in AMR gene profiles between these subgroups. We omitted genes that were ubiquitous throughout the phylogeny, as well as genes that were only present in three or fewer genomes, unless they were Bangladeshi *S*. Java genomes. The full gene matrices can be found in Tables S4–S6. BAPS clusters, geographical region and sample source are also depicted by the colour strips (see colour legend). The tree scale bar indicates the estimated mean number of nucleotide substitutions per site.

Next, we examined AMR and plasmid replicon gene distribution among the *S*. Paratyphi B complex (Fig. 5). All allelic variants of AMR genes including *gyrA* point mutations and plasmid replicons detected in the 271 genomes are summarized in Tables S5 and S6. The majority of genotypic antimicrobial resistance in *S*. Java was encoded by genomes in the animal-associated clade (PG10/BAPS cluster 1.7; Fig. 5). Besides that, we observed a stark lack of evidence for widespread extrinsic resistance gene and plasmid acquisition in the human-associated clades of the *S*. Paratyphi B complex, particularly in the Bangladeshi *S*. Java isolates. Three (3.9 %) Bangladeshi *S*. Java genomes carried *mphA, qnrB, bla*_DHA-7_ and *sul1* genes, which are predicted to confer resistance to macrolide, fluoroquinolone, beta-lactam and sulfonamide antibiotic classes, respectively (Fig. 5) [40]. Additionally, two of these isolates also carried *mphE* and *msrE,* encoding resistance to macrolide or erythromycin and streptogramin, respectively [40]. These gene sets were distinct from those seen in global *S*. Java isolates (*n*=10) carrying *bla*_CARB_, *aadA*, *floR* and *sul1* genes (Fig. 5), which are predicted to confer resistance to beta-lactam, aminoglycoside, chloramphenicol and sulfonamide, respectively.

While some of the Bangladeshi *S*. Java isolates harbouring AMR genes also carried plasmids, these AMR gene sets could not be co-located on the same contigs as the rep genes that define the plasmid Inc groups, due to fragmentation of the assemblies. Among the plasmid Inc types we found in the Bangladeshi *S*. Java population were one IncFIB (pHCM2), two IncI and three IncFII (S), IncFIB plasmids (Fig. 5). These plasmids generally encoded putative genes related to DNA metabolism and replication rather than virulence-associated determinants and AMR genes [51, 52], which could provide another explanation as to why we did not find AMR genes co-located with rep genes.

## Discussion

The serovar *S*. Paratyphi B is a source of confusion as biotype *sensu stricto* is a cause of invasive paratyphoid fever, while biotype Java is associated with non-invasive gastroenteritis. In this study, we coupled an existing nationwide enteric disease surveillance study across Bangladesh with a WGS approach to investigate the genomic epidemiology of *S. enterica* serotype Paratyphi B complex. Our surveillance showed that, in Bangladesh, the prevalence of *S*. Paratyphi B isolates is low (0.36%) compared to other enteric pathogens in this surveillance study [14]. Moreover, where previous studies have only reported serotyping results of *S*. Paratyphi B strains [53], this is the first study in Bangladesh to distinguish between *S*. Java and *S*. Paratyphi B *sensu stricto* biotypes, using WGS to confirm that *S*. Java is the variant responsible for the diarrhoeal disease in Bangladesh. This is in line with previous reports in which *S*. Java, not *S*. Paratyphi B *sensu stricto*, is the aetiological agent of non-invasive gastroenteritis [2–4, 6], and fits with the clinical characteristics displayed by patients in our study.

All except two of the Bangladeshi *S*. Java isolates clustered with isolates from the UK. This may be evidence of intercontinental long-range transmission; however**,** this is more likely explained by the lack of genome sequencing for isolates within this complex, and provides support for continued multi-pathogen genomic surveillance efforts. Our data show that *S*. Java is a globally relevant enteric pathogen in both high- and low-income settings, with clear signs of recent population expansions in clinically relevant lineages. The two *S*. Java lineages present in Bangladesh were linked with three STs, the most prevalent of which, ST43, is a globally distributed ST; seen in Singapore, Indonesia, India, Bangladesh, Malaysia, France, Finland, Germany, Spain, the UK and Canada (Fig. 2). On the other hand, STs 1577 and 2113 were only observed in Bangladesh and the UK. In addition, STs 43 and 1577 were reported previously in both poultry- and human-associated *S*. Java isolates in Bangladesh [12]. The finding of the same STs in human and animal *S*. Java isolates in Bangladesh suggests that further sampling and WGS of a variety of sources could provide insights into reservoirs of global STs, with WGS data providing higher discriminatory power for lineage placement than MLST alone. While our WGS study allowed us to describe the population dynamics of *S*. Java in Bangladesh, and identify in what proportions globally distributed or endemic STs are present, MLST is a lower-cost alternative to genomic surveillance and such information will facilitate long-term tracking of the population dynamics, supported by genomic surveillance where possible.

While our phylogenetic analysis closely resembled that of Connor *et al.* [2], the addition of these genomes into the existing phylogeny resulted in some minor changes to the placement of genomes within the previously described PGs 3 and 4, which upon updated BAPS analysis, were merged together into what we have called here BAPS cluster 1.1. There are three main differences between our updated phylogeny and the one in Connor *et al.* [2] that would account for these discrepancies: first, we did not use genomes from additional *Salmonella* serovars; second, our tree is constructed from an alignment of SNPs relative to the reference genome *S*. Paratyphi B SPB7 (whereas theirs is constructed from an alignment of core gene SNPs); and last, our tree contains 79 *S*. Java isolates from Bangladesh – a country that until now has been under-represented for bacterial pathogen WGS studies [2]. The differences between these phylogenetic trees reflect the differences in the aims of the studies.

Despite widespread and unregulated mis-use of antimicrobials in Bangladesh, we were surprised to observe little evidence of horizontal gene transfer of AMR genes. This contrasts with earlier studies in Scotland, England, Wales and the Channel Islands – where antimicrobial stewardship is tighter – which reported a range of AMR spectra in both in *S*. Java-infected patients and poultry since 2000 [54]. The latter study, however, did not take a genomic approach, and hence it is unknown (a) which lineages these samples belonged to and b) on which genetic element they were encoded. These factors could help explain the discrepancy between their results and ours. Importantly, our findings are supported by a recent study reporting chromosomal integron class 2-mediated vertical AMR gene inheritance in the PG10 poultry-associated *S*. Java lineage but limited acquisition of AMR genes in human *S*. Java lineages (Fig. 5) [2, 55]. There are several possible explanations for the lack of AMR genes in Bangladeshi *S*. Java. First, in general, NTS infection is a self-limiting disease, mostly manifesting as gastroenteritis, and rarely requires antimicrobial therapy, although they are sometimes taken to reduce the acuteness of symptoms [56]. Second, plasmids, which are known to carry AMR genes and act as a mode of dissemination of resistance determinants in enteric bacteria, were found rarely in Bangladeshi *S*. Java. The AMR genes we did find in our dataset are generally chromosomally encoded [2]. Worryingly, three *S*. Java isolates carried fluoroquinolone resistance genes. Fluoroquinolone-resistant *Salmonellae* are on the World Health Organization (WHO) priority list of bacteria for which new antibiotics are urgently needed. These findings highlight selective pressure towards resistance in circumstances where control and antimicrobial stewardship are challenging.

While our sample size was relatively small, we are confident that it is representative of the prevalence and dynamics of *S*. Java in Bangladesh, having covered eight divisions and a 4-year timespan. However, due to the low sample numbers, neither the ST distribution across the sites, nor the relationship between STs with symptoms or severity, reached statistical significance. A further limitation of our study is the fragmentation of assemblies: this prevented us from confidently assigning AMR genes to plasmids. Future work could include long-read sequencing to address this. Lastly, we were unlikely to detect *S*. Paratyphi B *sensu stricto* as the surveillance study underpinning the genomics was targeted to enteric pathogens. However, *S*. Paratyphi B *sensu stricto* is very rare globally [4], and symptoms can include diarrhoea; future surveillance efforts to include blood samples will increase the likelihood of our detecting *S*. Paratyphi B *sensu stricto* if it is present in Bangladesh.

This study highlights the importance of developing genomic surveillance systems in all settings in order to answer changing patterns of disease both nationally and internationally. We have used this approach to characterize the polyphyletic population structure of *S. enterica* serotype Paratyphi B and resolve the confusion associated with the spectrum of clinical symptoms. Our study provides a framework for future hospital surveillance-based genomic epidemiology studies in low-income countries. Continued molecular-based surveillance incorporating both WGS and MLST approaches will provide further information that can be used to design and implement better diagnostic tests, hence facilitating treatment options, and informing public health interventions in poor resource settings such as Bangladesh.

## Supplementary Data

Supplementary material 1

Supplementary material 2
