# Supplementary material for: Genome-wide analysis provides a deeper understanding of the population structure of the Salmonella enterica serotype Paratyphi B complex in Bangladesh
Source: Microb Genom. 2021 Sep 22;7(9):000617. doi: 10.1099/mgen.0.000617 (PMC8715441; doi:10.1099/mgen.0.000617)
Supplement: Supplementary material 1 [file mgen-7-0617-s001.pdf]

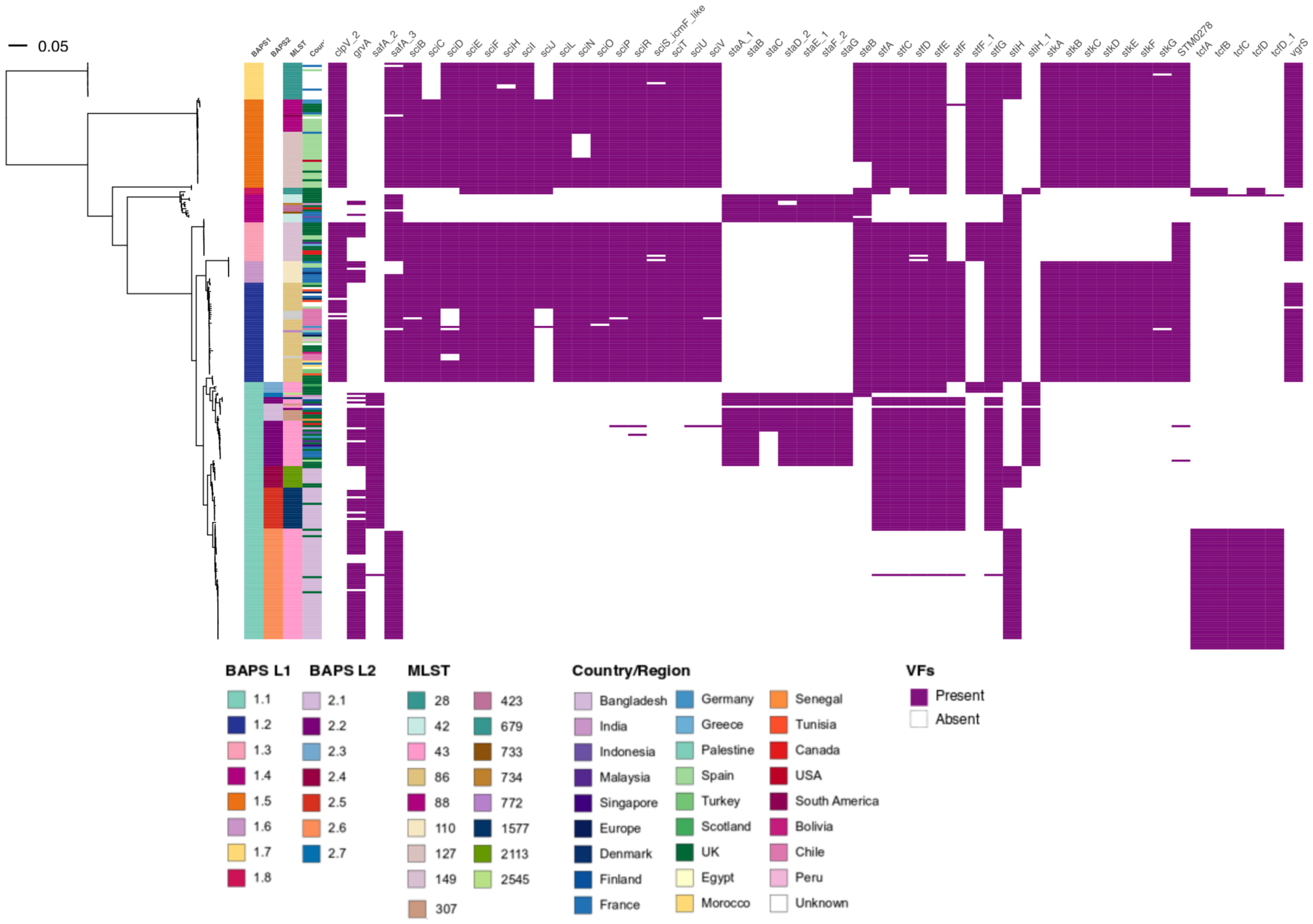
0.05
BAPS1
BAPS2
MLST
Coun
clpV_2
grvA
safA_2
safA_3
sciB
sciC
sciD
sciE
sciF
sciH
sciI
sciJ
sciL
sciN
sciO
sciP
sciR
sciS_icmF_like
sciT
sciU
sciV
staA_1
staB
staC
staD_2
staE_1
staF_2
staG
steB
stfA
stfC
stfD
stfE
stfF
stfF_1
stfG
stiH
stiH_1
stkA
stkB
stkC
stkD
stkE
stkF
stkG
STM0278
tcfA
tcfB
tcfC
tcfD
tcfD_1
vgrS
BAPS L1
1.1
1.2
1.3
1.4
1.5
1.6
1.7
1.8
BAPS L2
2.1
2.2
2.3
2.4
2.5
2.6
2.7
MLST
28
42
43
86
88
110
127
149
307
423
679
733
734
772
1577
2113
2545
Country/Region
Bangladesh
India
Indonesia
Malaysia
Singapore
Europe
Denmark
Finland
France
Germany
Greece
Palestine
Spain
Turkey
Scotland
UK
Egypt
Morocco
Senegal
Tunisia
Canada
USA
South America
Bolivia
Chile
Peru
Unknown
VFs
Present
Absent

**Fig. S1: Virulence factor distribution among the *S.* Paratyphi B complex:** A Maximum likelihood outgroup-rooted tree of 271 strains from the global collection, including Bangladeshi *S.* Java isolates from this study, alongside a presence/absence matrix of virulence factor genes for each isolate. Only genes with differential presence/absence patterns are depicted. BAPS clusters, MLST and country/region data are also depicted by the colour strips (see colour legend). The tree scale bar indicates the estimated mean number of nucleotide substitutions per site.

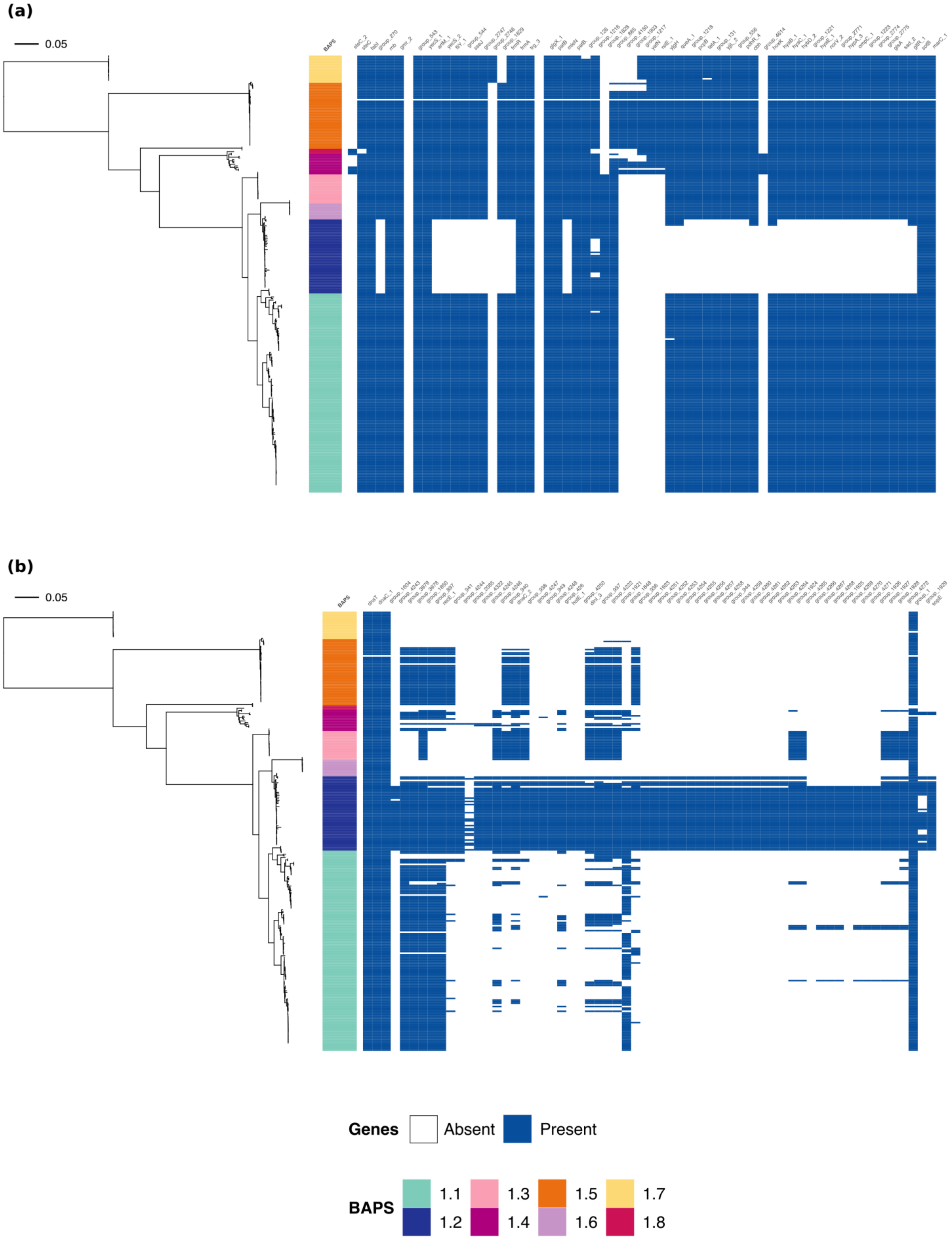
(a)
0.05
BAPS
(b)
0.05
BAPS
Genes
Absent
Present
BAPS
1.1
1.2
1.3
1.4
1.5
1.6
1.7
1.8

**Fig. S2: Genes specific and core to S. Paratyphi B sensu stricto (a) and S. Paratyphi B variant Java (b).** Gene presence and absence matrix plotted against the phylogeny of the *S*. Paratyphi B complex. Genes are ordered as determined by gene path analysis for *S*. Java, and by a representative assembly for *S*. Paratyphi B *sensu stricto*. Conservation of gene blocks, in separate loci, are shown by blue blocks, with flanking genes conserved by the whole complex also shown. BAPS clusters as determined in this study are also depicted by the colour strip adjacent to the tree. Genes in the gene block that are specific to that biotype but fall below the threshold to be defined as core to that biotype are also included.

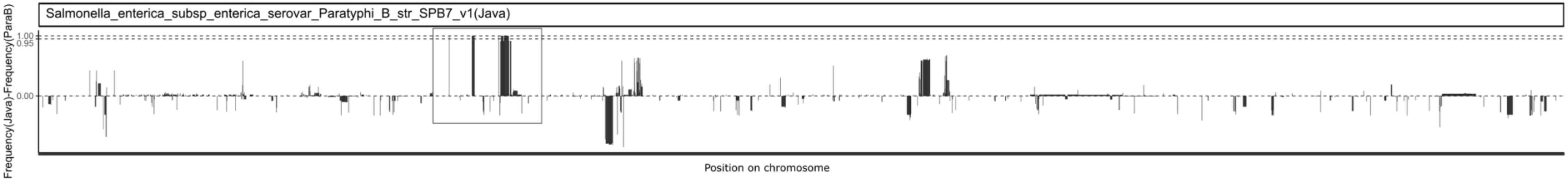


**Fig. S3: Frequency of *S*. Java-specific genes relative to their frequency in *S.* Paratyphi B *sensu stricto*.** The x axis shows the genes in their order on the chromosome of the reference genome *S*. Paratyphi B SPB7 (accession number CP000886). The difference in the frequency of each gene in *S*. Java relative to their frequency in *S*. Paratyphi B *sensu stricto* is plotted on the y axis, where genes with a frequency between 0.95 and 1.0 are specific to *S*. Java (i.e. they have a frequency of 0.95-1.0 in *S.* Java and 0 in *S*. Paratyphi B *sensu stricto*). Hence, *S*. Java-specific genes (in three loci, highlighted by the box) are shown by bars above the x axis. Genes with a frequency close to 0 are shared by both biotypes, and genes with a frequency < 0 are more common in *S*. Paratyphi B *sensu stricto* than *S*. Java, and hence are shown by bars below the x axis.

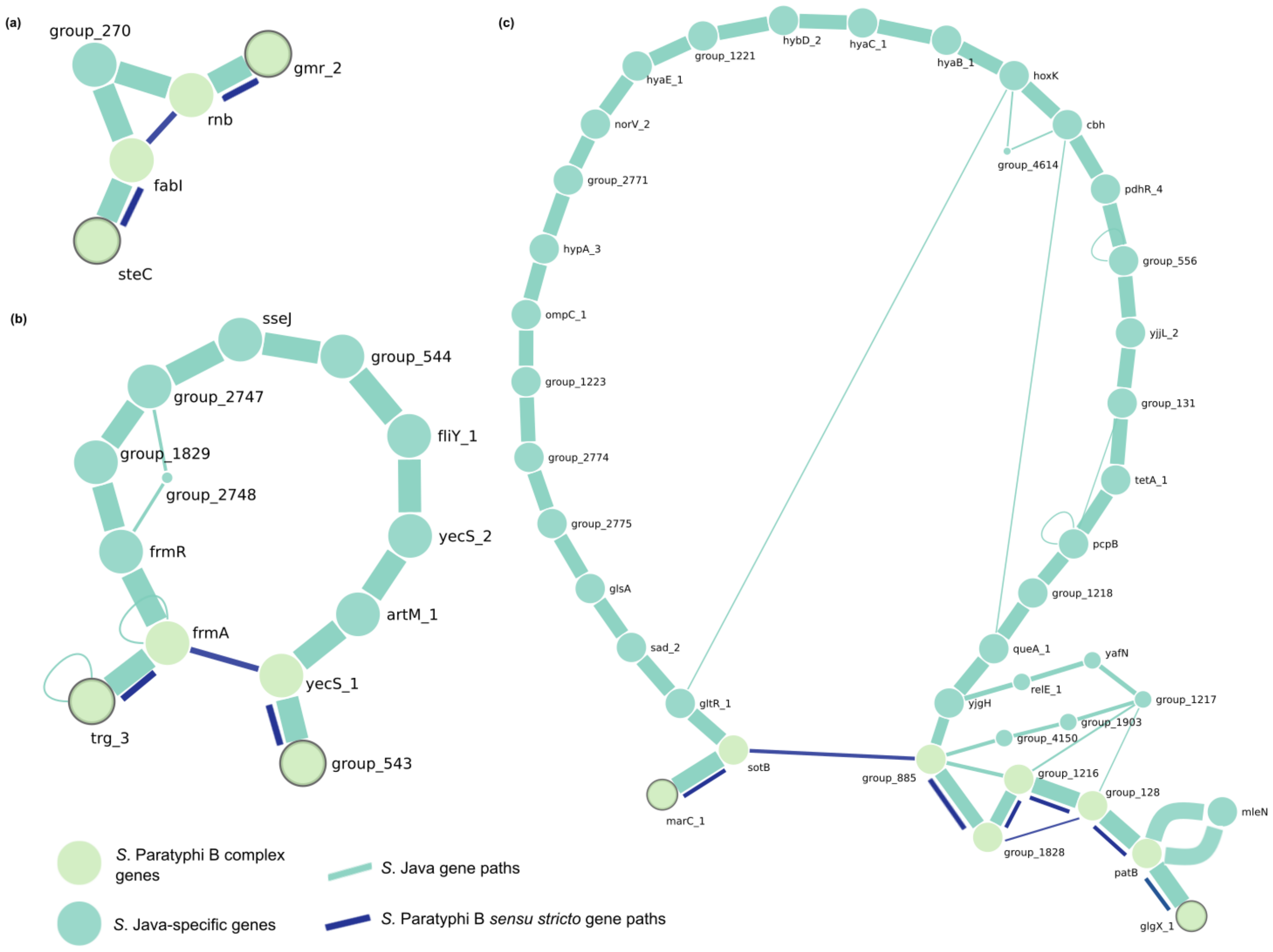


**Fig. S4: Gene paths and synteny analysis for *S*. Java-specific loci.** Genes corresponding to the three *S*. Java-specific loci (a, b, c) are depicted by coloured circles and paths are depicted by adjoining rectangles. The path/rectangle width is relative to the number of genomes that share that path. Genes and paths are coloured by biotype (see key). For each locus, conserved flanking genes are also included, outlined in grey.
